# Antioxidant Activity of the Essential Oil and Methanolic Extract of *Teucrium orientale* (L.) subsp. *taylori* (Boiss.) Rech. f.

**Published:** 2010

**Authors:** Hamzeh Amiri

**Affiliations:** *Department of Biology, Lorestan University, Khoramabad, Iran. *

**Keywords:** *Teucrium orientale *subsp. *Taylori*, Essential oil, Antioxidant activity

## Abstract

This study was designed to examine the chemical composition and *in-vitro *antioxidant activity of the essential oil and methanolic extract of *Teucrium orientale *subsp*. taylori*. The GC and GC–MS analysis of the essential oil resulted in determination of 40 components representing 96.4% of the oil. The major constituents of the oil were linalool (28.6%), caryophyllene oxide (15.6%), 1,8-cineol (4.5%), β-pinene (8.7%), 3-octanol (9.5%), β-caryophyllene (7.3%), and germacrene-D (4.1%). Antioxidant activities of the samples were determined by two different tests, namely DPPH and β-carotene- linoleic acid assay. In DPPH system, the weakest radical scavenging activity was exhibited by the non-polar sub fraction of methanolic extract (237.40 ± 2.1 μg/mL). Antioxidant activity of the polar sub fraction of methanolic extract was superior to all samples tested, showing an EC_50 _value of 61.45 ± 0.5 μg/ mL. The inhibition capacity (%) of the polar sub fraction of methanolic extract (95.21% ± 1.3) was found to be the strongest and almost equal to the inhibition capacity of the positive control BHT (94.9% ± 1.1). The amount of the total phenolics was the highest in the polar subfraction, *i.e. *370 μg/mg of the dry extract (37%). A positive correlation was observed between the antioxidant activity and the total phenolics of the extracts.

## Introduction

Oxidative stress by free radicals is an important event in cell that can cause aging and degenerative diseases including cancer, heart diseases, multiple sclerosis, Parkinson’s disease, autoimmune diseases and senile dementia. Stress, physical damage, viral infection and cytotoxic or carcinogenic compounds, as a consequence of chemical or biological aggression, may cause peroxidation of polyunsaturated fatty acids of cell membranes and liberation of toxic substances such as free radicals. Studies concerning the relationship between the mortality due to cancer and heart diseases, and consumption of fruits and vegetables indicated that polyphenols, being present in large amounts in fruits and vegetables, have a significant decreasing effect on the mortality rate from these diseases (1-3). On the other hand, oxidation of lipids, which occurs during raw material storage, processing, heat-treatment and storage of final products, is one of the basic processes causing rancidity of food products and their deterioration. Due to undesirable effects of oxidized lipids on human, it seems essential to decrease contact with products of lipid oxidation in food (4). In order to prolong the storage stability of foods, synthetic antioxidants are used in industrial processing. However, the side effects of some synthetic antioxidants used in food processing such as butylated hydroxytoluene (BHT) and butylated hydroxyanisole (BHA) have already been documented. For example, these substances can show carcinogenic effects in living organisms (5, 6). Therefore, authorities of government and consumers are concerned about the safety of the food products and the potential effects of synthetic additives on human health (7). 

Many species of fruits, vegetables, herbs, cereals, sprouts and seeds have been investigated for antioxidant activity during the past decade (8-11). Natural antioxidants are being extensively studied for their ability to protect organisms and cells from damage caused by oxidative stress which is considered as a cause of ageing and degenerative diseases (12). Herbs and spices are, in general, harmless sources for obtaining natural antioxidants. The antioxidant capacity of plants is clearly associated with the activity of ‘‘free radical scavenging enzymes’’ (superoxide dismutase, catalase, peroxidase, etc.) and the contents of antioxidant substances mainly phenolic compounds, carotenoids, tocopherol and ascorbic acid (13). There is an increasing demand to evaluate the antioxidant properties of plant extracts (14) and in recent years, attention has been focused on antioxidant products from natural sources.

*Teucrium *(from the Lamiaceae family) is a cosmopolitan genus of about 340 species and it has 13 species in Iran (15). *Teucrium *species are bitter, astringent, antirheumatic herbs that reduce inflammation, stimulate the digestion and have been used as herbal medicines for coughs and asthma since ancient times. Several studies about bacteriostatic, spasmolytic, antioxidant and antiinflammatory effects of *Teucrium *species have also been reported in the literature (16-19).

The present study deals with the chemical composition of the essential oil and the methanolic extract of *Teucrium orientale *(L.) subsp. *taylori *(Boiss.) Rech. f. and their antioxidative properties *.*

## Experimental

*Plant material*

*Teucrium orientale *subsp*. taylori *identified by Dr. Mozaffarrian, was collected during fruiting stage (15th Jun 2007) from Aleshtar, Lorestan province, Southwest of Iran. The voucher specimen was deposited at Herbarium of the Agriculture and Natural Resources Research Center of Lorestan Province, Khoramabad, Iran (no. 480). The collected plant materials were dried in the shade. 

*Isolation of the essential oil*

The dry aerial parts (with fruits) (100 g) of *T. orientale *subsp*. taylori *were hydrodistillated using a Cleavenger-type apparatus for 2 h followed by decanting and drying over anhydrous sodium sulfate.

*Preparation of the methanolic extract*

The air-dried and finely ground samples were extracted by the previously described method. Briefly, the sample weighing about 100 g was extracted in a Soxhlet with methanol (MeOH) at 60°C for 6 h. The extract was then filtered and concentrated *in-vacuo *at 45°C yielding a waxy material. The resulting extract (19.77% w/w) was suspended in water and partitioned with chloroform (CHCl_3_) to obtain water-soluble (polar) (13.21% w/w) and water-insoluble (non-polar) (4.55% w/w) subfractions, which were then lyophilized and kept in the dark at + 4 °C until used.

*GC analysis*

GC analysis was performed on a Shimadzu 15A gas chromatograph equipped with a split splitless injector and a flame ionization detector at 250 ºC.The N_2_ gas was used as a carrier gas (1 mL/min) and a DB-5 type column as the capillary column (50 m × 0.2 mm, film thickness 0.32 μm). Temperature within the column was kept at 60 ºC for 3 min, followed by an increase at a rate of 5 ºC min until it reached 220 ºC and maintained for 5 min. The relative percentages were calculated from the peaks’ areas using a shimadzu C-R4A chromatopac without applying correction factors.

*GC-MS analysis*

GC-MS analysis was performed on a Hewlett-Packard 5973 system with HP 5MS column (30 m × 0.25 mm, film thickness 0.25 μm). The column temperature was kept at 60 ºC or 3 min and programmed to reach 220ºC at a rate of 5ºC/min and stayed steady at 220ºC for 3 min. The components of the oil were then identified by comparison of their mass spectra and retention indices (RI) with those given in literature and those of the authentic samples (20).

*Antioxidant activity*

*DPPH assay*

The hydrogen atoms or electrons donation ability of the corresponding extracts and some pure compounds were measured by bleaching the purple colored methanolic solution of DPPH. The effects of methanolic extract and essential oil on DPPH radicals were evaluated according to a method described elsewhere (21). 4 mL samples of various concentrations of the extracts in methanol were separately added to a 1 mL solution of DPPH radical in methanol (final concentration of DPPH was 0.2 mM). The mixture was shaken vigorously and allowed to stand for 30 min after which the absorbance of the resulting solution was measured at 517 nm with a spectrophotometer (Shimadzu UV-1240, Kyoto, Japan). Inhibition of free radical DPPH as percentage [I(%)] was calculated as follows:

*I (*%) = 100 × (*A*_blank_ -*A *_sample_) / *A*_blank_

where *A*_blank_ is the absorbance of the control (containing all reagents except the test compound) and *A*_sample_ is the absorbance of the test compound. EC_50_ value (μg /mL) is the effective concentration at which DPPH radicals are scavenged by 50%. This was obtained by interpolation and using linear regression analysis. BHT was used as a control. 

*β-Carotene–linoleic acid assay*

In this assay, antioxidant activity was determined by measuring the inhibition of the volatile organic compounds and the conjugated diene hydroperoxides arising from linoleic acid oxidation (22). A stock solution of β-carotene–linoleic acid mixture was prepared as follows: 0.5 mg β-carotene was dissolved in 1 mL of chloroform (HPLC grade); 25 μL linoleic acid and 200 mg Tween 40 was added. Chloroform was completely evaporated using a vacuum evaporator. Then 100 mL of aerated distilled water was added with vigorous shaking; 2500 μL of this reaction mixture was transferred to test tubes and 350 μL portions of the extracts prepared in ethanol (at a concentration of 2 g/L) were added and the emulsion system was incubated for up to 5 h at 50°C. The same procedure was repeated with the positive control BHT and a blank. After this incubation period, absorbance values of the mixtures were measured at 490 nm. Antioxidant activities of the extracts were compared with those of BHT at the same concentration and the blank consisting only 350 μL ethanol.

*Assay for phenolic compounds*

The total phenolic constituents of the aforesaid extracts of *T. orientale *subsp*. tylori *were determined by previously reported methods involving Folin-Ciocalteu reagent and gallic acid as the standard (23, 24). The extract solution (0.1 mL) containing 1000 μg of the extract was transferred to a volumetric flask; 46 mL of distilled water and 1 mL Folin-Ciocalteu reagent were added and the flask was thoroughly shaken. After 3 min, 3 mL of a solution of 2% Na_2_CO_3 _were added and the mixture was allowed to stand for 2 h with intermittent shaking, after which the absorbance was measured at 760 nm. The same procedure was repeated for all the standard gallic acid solutions (0- 1000 mg 0.1 mL^-1^) and a standard curve was obtained with the equation given below:

Absorbance = 0.0012 × gallic acid (μg) + 0.0033

## Results and Discussion

Hydrodistillation of the dried flowering aerial parts of *T. orientale *subsp. *taylori *gave 0.7% (v/w) of a yellowish oil. 

The chemical composition of the essential oil of *T. orientale *subsp. *taylori *was determined by GC and GC–MS (Table 1). As shown in Table 1, forty components were identified, accounting for 96.4% of the total oil composition. The major constituents were linalool (28.6%), caryophyllene oxide (15.6%), 3-octanol (9.5%), β-caryophyllene (7.3%), 1,8-cineole (4.5%), and germacrene-D (4.1%). The main monoterpene component was linalool (17.0%), which was also seen in *T. oxyliepis *and *T. asiaticum *as the main component. β-Caryophyllene (7.3%) and caryophyllene oxide (15.6%) were the main sesquiterpenes in the oil, which were observed in many other species of *Teucrium *(25).

**Table 1:** Chemical composition of the essential oil of *Teucrium orientale *subsp. *taylori*

**No. **	**Compounds **	**RI **	**Percentage **	**NO. **	**Compounds **	**RI **	**Percentage **
1	(*E*)-2-Hexanal	854	0.1	21	Linaloyl acetate	1258	0.6
2	α-Thujene	929	0.2	22	Bornyl acetate	1289	0.1
3	α-Pinene	939	2.1	23	Eugenol	1353	0.3
4	Banzyl aldehyde	959	0.1	24	α-Copaene	1372	0.6
5	β-Pinene	974	8.7	25	β-Burbonene	1382	0.4
6	Sabinene	988	0.2	26	β-Cubebene	1388	0.5
7	3-Octanol	993	9.5	27	α-Cedrene	1408	0.2
8	Limonene	1025	0.4	28	β-Caryophyllene	1419	7.3
9	1,8-Cineol	1031	4.5	29	α-Bergamotene	1434	1
10	γ-Terpinene	1060	0.1	30	α-Humulene	1449	0.7
11	Linalool	1097	28.6	31	Germacrene-D	1480	4.1
12	*n*-Nonanal	1102	0.2	32	β-Bisabolene	1506	3.4
13	*p*-2-Menthen-1-ol	1113	0.3	33	δ-Cadinene	1526	1.2
14	α-Campholenal	1125	0.4	34	Elemol	1550	1.2
15	(*E*)-Pinocarveol	1134	0.6	35	Caryophyllene oxide	1596	15.6
16	(*E*)-Verbenol	1140	0.2	36	α-Cedrol	1599	0.2
17	Borneol	1169	0.2	37	α-Cadinol	1656	0.2
18	Terpinene-4-ol	1175	0.2	38	Banzyl banzoate	1762	0.5
19	Myrtenal	1196	0.4	39	Hexadecanoic acid	1972	0.4
20	(*E*)-Carveol	1219	0.1	40	Phytol	2114	0.8

Caryophyllene oxide (33.5%), linalool (17.0%) and β-caryophyllene (9.3%) were also identified as the major constitutes of the oil of *T. orientale *subsp. *orientale *collected from Fars province, Iran (26). On the other hand, β-caryophyllene (21.7%) was reported as the most abundant component of the oil of *T. orientale *var. *puberulens *(27). Comparison between the analysis results from the oil of *Teucrium orientale *subsp*. taylori *in this research and the other studies on *Teucrium orientale *subsp*. orientale *showed that the main components of the oils of the two subspecies are similar with a few differences in percentages of the major constituents. 

In general, β-caryophyllene and caryophyllene oxide were reported as the main sesquiterpenes in many of the *Teucrium *species (28). 

To the best of our knowledge, the essential oil of *T. orientale *subsp. *taylori *has not been the subject previously studied. 

*Antioxidant activity*

Antioxidant activities of the essential oil and subfractions of the methanolic extract of *Teucrium orientale *subsp*. taylori *were determined by two different test systems namely DPPH and β-carotene-linoleic acid. All the data are presented in Table 2.

**Table 2:** Antioxidant activities of the essential oil and methanolic subfractions of *Teucrium orientale *subsp*. taylori* ^a^

**Sample**	**DPPH** **b**	**β-carotene/linoleic acid** **c**
Oil	121.60 ± 0.7	79.85 ± 1.4
Polar subfraction	68.45± 0.5	77.40 ± 0.9
Nonpolar subfraction	237.40 ± 2.1	95.21 ± 1.3
BHT	16.8 ± 0.6	94.90 ± 1.1

^a^ Results were reported as the mean values of three different experiments.

^b^ EC_50 _values of DPPH assay (as μg/mL).

^c ^Given as percentage (%) of inhibition of the linoleic acid.

In DPPH method, the antioxidants react with the stable free radical. 1,1-diphenyl-2-picrylhydrazyl (deep violet color) and convert it to 1,1-diphenyl-2-picrylhydrazine with discoloration. The degree of discoloration indicates the free radical scavenging activities of the sample/antioxidant and it has been found that the known antioxidants such as cysteine, glutathione, ascorbic acid, tocopherol, and polyhydroxy aromatic compounds (hydroquinone, pyrogallol, etc.) reduce and decolorize 1,1-diphenyl-2-picrylhydrazyl by their hydrogen donating ability (29). In the present study, the polar sub-fraction of methanolic extract was able to reduce the stable radical DPPH to 1,1-diphenyl-2-picrylhydrazine with an IC_50_ value of 68.45 ± 0.5 μg/mL . Also, the essential the oil and non-polar subfraction of methanolic extract showed activity with an IC_50_ of 121.60 ± 0.7 μg/mL and 237.4 ± 2.1 μg/mL, respectively. Considering the free radical scavenging activity, the superiority of the polar sub fraction of methanolic extract could be attributed to the presence of phenolic compounds as they comprise 37% of the extract. The synergistic effects of phenolic acids (e.g., rosmarinic acid) and polyphenols, as well as other chemicals such as flavonoids could also responsible for the radical scavenging activity observed in methanolic extracts (30). 

In β-carotene-linoleic acid model system, β-carotene undergoes rapid discoloration in the absence of an antioxidant. This is because of the coupled oxidation of β-carotene and linoleic acid, which generates free radicals. The linoleic acid free radical formed upon the abstraction of a hydrogen atom from one of the diallylic methylene groups attacks the highly unsaturated β-carotene molecules. As a result, β-carotene is oxidized and broken down in part; subsequently the system loses its chromophore and characteristic orange color, which is monitored spectrophotometrically. As can be seen from Table 2, the percent inhibition capacity of the polar subfraction of methanolic extract (95.21% ± 1.3) was found to be superior to all samples, being almost equal to the inhibition capacity of the positive control BHT (94.50% ± 1.8). This was followed by the essential oil. Non-polar subfraction of *T. orientale *subsp*. taylori *essential oil showed the weakest activity potential in this test system.

The auto-oxidation of linoleic acid in the absence of the volatiles and methanolic extracts accompanies the rapid increase of peroxides. According to Farag *et al*. , there is a relationship between the inhibition of the hydroperoxide formation and the presence of some phenolic nucleus in essential oils and extracts (31). The antioxidative properties in natural sources have been reported to be mostly due to phenolic compounds (32).

The antioxidant properties of the essential oil and different extracts of *T. orientale *var. *orientale *have been previously reported (33). According to Cakir *et al*., the antioxidative activities of the extracts of *T. orientale *var. *orientale *obtained by polar organic solvents (acetone and methanol) were also greater than those of the extracts obtained by non-polar organic solvents (chloroform and petroleum ether) (33). Hence, it can be suggested that the polar compounds are mainly responsible for the antioxidant activity. Similarly, the methanol and acetone extracts of the plant samples harvested at all three stages exhibited the highest DPPH radical scavenging activity. In addition, antioxidant activities of the essential oils of *T. orientale *var. *orientale *from different harvesting stages have also previously been reported and the steam distillation oils from the budding and flowering stages showed the highest antioxidant activities (19).

Reviewing the related literature shows that other species of *Teucrium *such as *T. montanum, T. chamaedrys, T. polium*, *T. marum *and *T. sauvagei *have also antioxidant activity (34).

## Conclusion

In order to prolong the storage stability of foods and to reduce the damage to human body, synthetic antioxidants are used in industrial processing. However, the side effects of some sythetic antioxidants as butylated hydroxyanisole (BHA) and butylated hydroxytoluene (BHT) have already been documented. For example, these substances can show carcinogenic effects in living organisms (5, 6). Therefore, governmental authorities and consumers are concerned about the safety of the food products and the potential effects of synthetic additives on human health (7). Compared to the antioxidant activity of the standard compound used in this study (BHT), polar subfraction of the methanolic extract of *T. orientale *subsp. *tylori *exerted strong antioxidant effect, which was almost equal to BHT. 

In conclusion, the results presented here can provide evidence that the polar sub fraction of the studied methanolic extract could be used in food industries and other fields, which process natural products. However, further studies are certainly needed for a better clarification of the potential cyctotoxicity and other biological effects of the plant species presented here.
